# Muscle characteristics in chicks challenged with *Salmonella* Enteritidis and the effect of preventive application of the probiotic *Enterococcus faecium*

**DOI:** 10.3382/ps/pey561

**Published:** 2018-12-26

**Authors:** R Zitnan, E Albrecht, C Kalbe, C Miersch, V Revajova, M Levkut, M Röntgen

**Affiliations:** 1 National Agriculture and Food Centre, Research Institute of Animal Production, Nitra, Kosice, Slovakia; 2 Institute of Muscle Biology and Growth, Leibniz Institute for Farm Animal Biology (FBN), Dummerstorf, Germany; 3 Department of Pathological Anatomy, University of Veterinary Medicine and Pharmacy, Kosice, Slovakia

**Keywords:** Muscle, *Salmonella*, probiotic, *Enterococcus*, broiler

## Abstract

The present study was conducted to assess the effects of the probiotic *Enterococcus faecium* AL41 (**EF**) and of the enteric pathogen *Salmonella* Enteritidis PT4 (**SE**) on the development of posthatch pectoralis major muscle (**PM**) of broiler chicks. The four experimental groups were control (**CON**), EF, SE, and EF*+*SE (EFSE). EF and SE were given *per os* from days 1 to 7 and at day 4 posthatch, respectively. Muscle samples from 6 chicks per group were taken at day 8 (D8) and day 11 (D11) to evaluate PM myofiber growth, capillarization, DNA, RNA, and protein content, as well as enzyme activities (isocitrate dehydrogenase, lactate dehydrogenase, creatine kinase). PM growth rate was 7.45 ± 2.7 g/d in non-SE groups (CON, EF) and 5.10 ± 1.82 g/d in SE-infected groups (*P* < 0.02). Compared with group CON, application of bacteria (groups EF and SE) reduced the fiber cross-sectional area (246 and 262 vs. 347 ± 19 μm^2^) and the number of myonuclei per fiber (0.66 and 0.64 vs. 0.79 ± 0.03). At D11, hypertrophic myofiber growth normalized in the EF group, but negative effects persisted in SE and EFSE birds contributing to lower daily PM gain. In addition, SE infection strongly disturbed PM capillarization. Negative effects on capillary cross-sectional area and on the area (%) covered by capillaries persisted until D11 in the SE group, whereas pre-feeding of EF restored capillarization in the EFSE group to control levels. We conclude that supplementation of the probiotic bacteria EF AL41 had positive effects on PM capillarization and, thus, on delivery of O_2_, supply of nutrients, and removal of metabolites. Supplementation of probiotic bacteria might therefore reduce energetic stress and improve muscle health and meat quality during SE infection.

## INTRODUCTION

*Salmonella enterica* ser. Enteritidis (**SE**) is a facultative intracellular pathogen that can cause disease in animals and in man. In poultry, SE infection causes significant production losses, reduces welfare of birds, and increases the risk of contamination of poultry products for human consumption (Sackey et al., 2001; Pan and Yu, 2014; Iheukwumere et al., 2017). Poultry meat and eggs are considered to be the major source of infection for humans, and antibiotic-resistant strains have been isolated from carcass, drinking water, and litter (Sackey et al., 2001; Heyndrickx et al., 2002; Kilonzo-Nthenge et al., 2008; Velasquez et al., 2018).

Salmonellae are not native members of the intestinal microbiota, but early posthatch chicks, which have an immature immune system and have not yet established a stable gut microflora (Jeurissen and Janse, 1996; Crhanova et al., 2011; Pan and Yu, 2014), are highly susceptible to SE colonization. After oral infection of chicks with SE and its initial multiplication in the gut lumen, the pathogens attach to and enter epithelial cells of the intestinal villi, where they can survive and multiply (Rychlik et al., 2014). At the local level, SE infections lead to the alteration of the gut microbiota and damage to the intestinal barrier accompanied by adverse effects on digestion, malabsorption of nutrients, and activation of the host's immune compartment leading to inflammation (Rychlik et al., 2014). Chick macrophages and heterophiles are the first line of defense and are recruited in response to chemokines released by infected enterocytes (Kogut et al., 1994; van der Heijden and Finlay, 2012). Interestingly, the inflammatory process starts to be downregulated at 4 D after SE infection when anti-inflammatory cytokines (IL-10, tumor necrosis factor-ß) and the number and function of regulatory T cells are upregulated (Shanmugasundaram et al., 2015; Kogut et al., 2016). Because of the resulting SE tolerance, the pathogens often persist in the host's intestinal tract (particularly in the caeca) without visible clinical signs (Herich et al., 2010). Carrier birds excrete the bacteria intermittently and constitute an important source of infection (Ducatelle et al., 2000; Kogut et al., 2016; Velasquez et al., 2018). Salmonellae also colonize the Peyer's patches, from which they can disseminate into the liver and spleen (Monack et al., 2000; Crhanova et al., 2011); these organs are the sites of intracellular proliferation and the spreading of the bacteria causing systemic diseases with the translocation and dissemination of toxins and bacteria to various internal organs (Vasquez-Torres et al., 1999; Levkut et al., 2009; Herich et al., 2010; Iheukwumere et al., 2017).

A dense and complex population of commensal bacteria is an important factor with regard to protection from enteric infection (Pan and Yu, 2014), but its development is delayed in chicks hatched in incubators without contact to hens (Crhanova et al., 2011; Biloni et al., 2013). One non-antibiotic possibility for reducing intestinal colonization and for lowering the shedding of SE is the application of protective microflora (Ribeiro et al., 2007; Téllez et al., 2015) such as probiotics (live microbial feed supplements), which have been shown to compete with pathogens for nutrients and adhesion receptors (“competitive exclusion”) (Audisio et al., 1999; Mead, 2000; Al-Khalaifah, 2018). Probiotics also produce antibacterial substances (van der Wielen et al., 2002; Levkut et al., 2009), and stimulate the production of mucus and specific antibodies to modulate the local immune response (Forder et al., 2007; Levkut et al., 2012; Yang et al., 2012). In addition, positive effects on poultry growth performance and on feed utilization efficiency have been reported for various probiotics (Al-Khalaifah, 2018) and shown to be associated with enhanced gut barrier function, the stimulation of posthatch intestinal development, and improved gut morphology (Awad et al., 2009; Flint and Garner, 2009; Biloni et al., 2013). The species *Enterococcus faecium* (**EF**) possesses probiotic properties and reduces the colonization and proliferation of enteric bacteria including SE (Audisio et al., 1999; Herich et al., 2010; Lauková et al., 2012; Téllez et al., 2015). EF strains are able to produce bacteriocins, which, in contrast to antibiotics, have a relative narrow killing spectrum and are toxic only to bacteria closely related to the producing strain (Riley and Wertz, 2002; Lauková et al., 2003; Lauková et al., 2012). In chicken, preventive early application of EF has been shown to decrease cecal colonization with pathogenic SE, to promote the development of the small intestine and its protection barrier (Herich et al., 2010; Ševčíková et al., 2016), and to stimulate innate and acquired immune responses (Levkut et al., 2012; Dina and Hams, 2016).

In addition to health problems, SE infection induces economic losses by reducing growth performance (ADG, gain to feed ratio) and leads to increased production costs mainly via reduced feed conversion rate (Abdel Hamid et al., 2013; Dina and Hams, 2016). Inbred chicken strains selected for meat production (broilers) show accelerated muscle growth and, in particular, enhanced growth of the pectoralis muscle (**PM**), an important component of posthatch BW gain (Aberle and Stewart, 1983; Scheuermann et al., 2003; Collins et al., 2014; Geiger et al., 2018). Muscle fiber number is established at hatch (Stockdale and Miller, 1987), and therefore, muscle growth in chicken mainly results from muscle hypertrophy occurring by accretion of protein and new myonuclei that originate from so-called satellite cells (**SC**) (Aberle and Stewart, 1983; Duclos et al., 2006; Jaquemin et al., 2007). Most adult SC are quiescent and localized adjacent to myofibers in a niche underneath their basement membrane (Campion, 1984). In growing broiler muscles, a higher percentage of SC proliferates with maximum activity between days 2 and 4 after hatching, and after differentiation, most of them fuse to existing myofibers to enlarge their diameter (Halevy et al., 2000; Berri et al., 2006; Duclos et al., 2006). Thus, the early posthatch period is the most important period for maintaining SC activity and subsequent muscle growth (Mozdziak et al., 1997; Harthan et al., 2013). The SC myogenic program is primarily mediated by myogenic transcription factors (Yablonka-Reuveni and Paterson, 2001; Halevy et al., 2004). However, SC functionality is also sensitive to the cellular environment and influenced by various growth factors, cytokines, and pathogen-associated molecules (Erbay et al., 2003; Haddad et al., 2005; Jaquemin et al., 2007; Frost and Lang, 2008; Velleman et al., 2010). Like immune cells, skeletal muscle fibers and myogenic cells express Toll-like receptors and, thus, can recognize and react directly to bacterial cell wall components such as lipopolysaccharide (**LPS**) and lipopeptides (Lang et al., 2003; Frost et al., 2006). LPS has been shown to induce metabolic changes and oxidative stress and to enhance the expression of pro-inflammatory mediators (Frost et al. 2002; Lang et al., 2003; Nunes et al., 2005), all of which contribute to negative effects of pathogens on skeletal muscle development (Cooney et al., 1999; Frost et al., 2003; Strle et al., 2006; Whitlock et al., 2008; Al-Shanti et al., 2014).

We hypothesize that systemic effects of SE colonization and of the associated immune challenge include negative consequences on histomorphological and biochemical characteristics of muscle tissue thereby contributing to reduced muscle growth. In the present study, we have assessed effects of the probiotic bacterial strain EF AL41 or of an infection with SE on early posthatch PM development of chicks. We have investigated processes of myofiber growth and capillarization, muscle tissue content of DNA, RNA, and protein, and muscle enzyme activities. In addition, we have evaluated whether a one-week application of EF helps to circumvent or prevent possible negative consequences of SE infection on muscle growth and characteristics.

## MATERIAL AND METHODS

### Animals, Experimental Design and Sample Preparation

*Salmonella*-free one-day-old chicks (n = 220) of Cobb-500 hybrids were randomly divided into 4 groups (N = 55). Chicks of each group were randomly assigned to one of three pens (2 × 18 and 1 × 19 chicks) with a floor-covering of cellulose cotton (Hartmann, Bratislava, Slovak Republic) for the 11-D experimental period, and each pen was considered as one replicate. Bedding was changed daily during the course of the experiment. Chicks were reared under a lighting regime of 23 h light and 1 h dark. According to the requirements of the chick`s age group, the room temperature was 32°C during the first week and then was reduced by 2°C with each successive day. Relative humidity lay within a range of 50 to 60%. Birds had free access to feed (commercial BR1 starter diet, Barbara Company, Čaňa, Slovak Republic) and drinking water. Appropriate cleaning and feeding regimes were used to prevent cross-contamination throughout the experiment. The chicks were kept in the menagerie of the Department of Pathological Anatomy, University of Veterinary Medicine and Pharmacy, Košice, Slovakia (SK P 52,004), in accordance with the rules and approval of the Ethics Committee, and the experiments were authorized by the Committee for Animal Welfare of the Ministry of Agriculture of the Slovak Republic (permission number 2730/13–221).

The four experimental groups were control (**CON**), *Enterococcus faecium* AL41 (EF), *Salmonella* Enteritidis PT4 (SE), and combined EF*+*SE (**EFSE**). Throughout the experiment, control birds were fed with the commercial diet only without application of any bacteria (negative control). From day 1 to day 7 of the experiment, the probiotic bacterial strain *Enterococcus faecium* AL41 (provided by Dr. Andrea Lauková, Institute of Animal Physiology, Slovak Academy of Science, Košice, Slovakia) was administered *per os* at a dosage of 10^9^ colony-forming units (**CFU**) in 0.2 ml PBS to chicks of the EF and EFSE groups. Experimental infection of the SE and EFSE groups was carried out individually on day 4 of the experiment. SE was kindly provided by Dr. František Šišák from Veterinary Research Institute, Brno, Czech Republic. The bacterium was isolated from liver and caecum of one-day-old chicks and cultured for 20 h in brain heart infusion broth (Oxoid, Basingstoke, UK) at 37°C. Purity of the SE culture was tested by plating on blood agar and xylose-lysine-deoxycholate agar (Oxoid, Basingstoke, UK). The number of CFU was counted after plating ten-fold dilutions of the bacterial suspension on xylose-lysine-deoxycholate agar. To prepare the inoculum, 10^8^ CFU per chick were suspended in 0.2 ml PBS and given in a single dose *per os*.

At day 4 (day 8 of life; **D8**) and day 7 (day 11 of life; **D11**) after infection or sham infection (CON and EF, 0.2 ml PBS) with salmonellae, 6 chicks per group (2 chicks per pen) were randomly selected and killed by combined intraperitoneal injection of xylazine and ketamine. Commercially available solutions containing 2% xylazine (Rometar, SPOFA, Praha, Czech Republic) and 5% ketamine (Narkoman, SPOFA, Praha, Czech Republic) were used at dosages of 0.6 and 0.7 ml/kg BW, respectively. Thereafter, the pectoralis muscles were dissected immediately and weighed. Tissue samples from PM were excised for biochemical investigations and histology as described by Scheuermann et al. (2004), cut into pieces of about 0.5 × 0.5 × 1.0 cm, and immediately fixed in liquid nitrogen. They were then stored at −80°C until further preparation.

### Biochemical Analyses

Frozen PM samples (100 mg/per chick) were powdered under liquid N_2_ by using a mortar and pestle, extracted in a 1:20 (wt/vol) dilution of 0.01 M potassium phosphate buffer containing 4.2 mM dipotassium phosphate, 5.8 mM monopotassium phosphate, 150 mM potassium chloride, and 1 mM EDTA (pH = 6.9), and homogenized with a tissue grinder (Potter Elvehjem, Wheaton Science Products, Milliville, NJ, USA). Muscle homogenates were centrifuged at 14 000 g (15 min, 4°C), and resulting supernatants were kept on ice. All enzyme activity assays and determination of protein content were conducted the same day.

#### Enzyme Assays and Protein Content

Specific activities of creatine kinase (**CK**, EC 2.7.3.2), isocitrate dehydrogenase (**ICDH**, EC 1.1.1.42), and lactate dehydrogenase (**LDH**, EC 1.1.1.28) were measured as described by Lösel et al. (2013). Briefly, CK activity was measured at 37°C in muscle homogenates (diluted 1:200) by using a commercial kit (Biomed, Oberschleissheim, Germany). LDH and ICDH activities were analyzed at 25°C according to modified assay protocols from Sigma (http://www.sigmaaldrich.com/life-science/metabolomics/enzyme-explorer/learning-center/assay-library.html). LDH activity was determined in muscle homogenates (diluted 1:15 for muscle samples from D8 or 1:30 for muscle samples from D11), whereas ICDH activity was determined in undiluted muscle homogenates. The protein content of muscle homogenates (diluted 1:80) was analyzed according to Peterson (1977). All enzyme activities and protein content were determined on a Spectramax Plus384 spectrophotometer/plate reader (Molecular Devices Corporation, Sunnyvale, CA, USA). Per animal, three technical replicates were performed.

#### DNA and RNA Content

The DNA content of muscle homogenates (diluted 1:8) was measured with the fluorescent dye Hoechst 33258 against a calf thymus DNA standard (Sigma-Aldrich GmbH, Steinheim, Germany) as mentioned in Rehfeldt and Walther (1997). The RNA content of muscle homogenates (diluted 1:80) was quantified fluorometrically with SYBR Green II against a calf liver RNA standard (Sigma-Aldrich GmbH, Steinheim, Germany) as published by Oksbjerg et al. (2000). DNA and RNA assays were performed in 96-well quartz microwell plates by using a Flx-800-I microplate fluorescence reader (Bio-Tek Instruments Inc., Bad Friedrichshall, Germany). Per animal, three and two technical replicates were performed for DNA and RNA, respectively.

### Histomorphological Analysis of Pectoralis Muscle

Serial transverse sections of 10 μm were cut at −20°C using a cryostat microtome Leica CM3050 S (Leica Microsystems, Wetzlar, Germany). Cross-sections were stained using standard protocols for hematoxylin/eosin (**HE**) for nuclei and cytoplasm (Romeis, 1989), and with eosin and alkaline phosphatase (**EAP**, Spannhof, 1967), for cytoplasm and capillaries, respectively. Digital images were acquired with a CC-12 high-resolution color camera (OSIS, Münster, Germany) by routine brightfield microscopy (Nikon Microphot SA, Nikon, Düsseldorf, Germany). Muscle structure and capillarization were then evaluated using a computerized image analysis system with CELL˄F software (OSIS). Per chick 3 randomly selected H/E stained slides were used as replicates. The number of muscle fibers and myonuclei was then analyzed in three random fields each covering an area of 64,613 μm^2^ in these slides. The total analyzed muscle area per animal was 0.194 mm². Incomplete muscle fibers from the upper and left edge of the image were included and from the lower and right edge were excluded. The ratio between counted numbers of myonuclei and muscle fibers was calculated as nuclei/fiber. The size of muscle fibers was an estimated muscle fiber cross sectional area (**FCSA**) determined by dividing the analyzed area by the number of muscle fibers. The macro program for capillarization included the following steps: loading the image, counting of muscle fibers, extraction of the red channel of the image, enhancing of contrast, setting the threshold for capillaries, interactive deletion of false detected areas (e.g., artifacts), and automatic measurement of capillary traits. Again, three random fields were analyzed per slide and animal. Capillary cross-sectional area (**CCSA**), capillary density (number per mm²), number of capillaries per muscle fiber, and the area percentage of capillaries were determined.

### Statistical Analysis

Statistical analyses were performed by using SigmaPlot 11.0 (Systat Software Inc.). All data are presented as least square means (**LSM**) with SEM. Statistical significance of data was assessed by two‐way ANOVA (with Holm Sidak's multiple comparison tests) as appropriate. The model included the fixed effects group (levels: CON, EF, SE, and EFSE) and age (levels: 8 and 11 D posthatching; D8 and D11) and the corresponding interaction group x age. A *P*-value of <0.05 was considered statistically significant.

## RESULTS

### Pectoralis Muscle Weight Gain

PM weight in all groups increased significantly from D8 to D11 (Table 1), but PM growth rate between D8 and D11 was negatively affected in groups infected with *Salmonella* Enteritidis (SE and EFSE). PM growth rate amounted to 7.45 ± 2.7 g/d in non-SE groups (CON, EF) and to 5.10 ± 1.82 g/d in SE-infected groups (*P* < 0.02).

**Table 1.** Age-dependent changes of weight, contents and concentrations of DNA, RNA, and protein, and enzyme activities in pectoralis muscle (PM) of chicks.

	Day 8	Day 11	*P* value	Group
PM weight, g	18.6±0.7	37.4±1.3	<0.001	
Total DNA, mg	3.0±0.1	5.6±0.2	<0.001	
Total RNA, mg	19.0±0.7	27.3±0.9	<0.001	
Total protein, mg	1164 ± 52	2689 ± 108	<0.001	
DNA/RNA	0.169±0.005	0.205±0.005	<0.001	
DNA, μg/g	165 ± 4	150 ±3	*0.006	EF, SE
RNA, μg/g	1046 ± 39	736 ± 17	<0.001	
Protein, mg/g	62±0.8	72±1,3	*<0.001	EF, SE, EFSE
DNA/protein, μg/mg	2.66±0.09	2.09±0.04	*<0.001	C, EF, SE
RNA/protein, μg/mg	16.9±0.7	10.3±0.3	<0.001	
protein/RNA, mg/μg	0.062±0.003	0.099±0.003	<0.001	
Total CK, IU	38,271±2357	98,289±4725	<0.001	
CK IU/g	2043±38	2621±69	<0.001	
CK/protein, IU/mg	32.8±0.5	36.7±1.1	*<0.001	SE
ICDH, IU/g	2.52±0.04	2.37±0.06	0.028	
LDH, IU/g	442±15	618±19	*<0.001	C, EF, SE
ICDH/protein,IU/mg	0.041±0.001	0.033±0.001	<0.001	
LDH/protein, IU/mg	7.09±0.23	8.64±0.28	*<0.001	C, EF, SE
LDH/ICDH	176±7	266±11	*<0.001	C, EF, SE

Values are least square means ± standard error.

CK, creatine kinase; ICDH, isocitrate dehydrogenase; LDH, lactate dehydrogenase.

*Marks significant age-dependent changes within distinct groups (C, control; EF, *Enterococcus feacium* AL41; SE, *Salmonella* Enteritidis PT4; EFSE, EF + SE).

### DNA, RNA, Protein and Muscle Enzymes

Results on age effects are summarized in Table 1. PM growth was accompanied by elevated total amounts of DNA, RNA, and protein at D11 compared with D8. In all groups, DNA/RNA ratio, CK total activity and concentration of PM increased between D8 and D11, whereas CK activity per mg protein increased in the SE group only. The concentration of RNA decreased with age, and correspondingly, the protein/RNA ratio was higher at D11 compared with D8. An age-dependent decrease of the DNA/protein ratio was observed in CON, EF, and SE groups, whereas no change was found in the EFSE group. ICDH activity decreased between D8 and D11. However, LDH activity and LDH/ICDH ratio increased in CON, EF, and SE groups only (Table 1).

**Figure 1.**
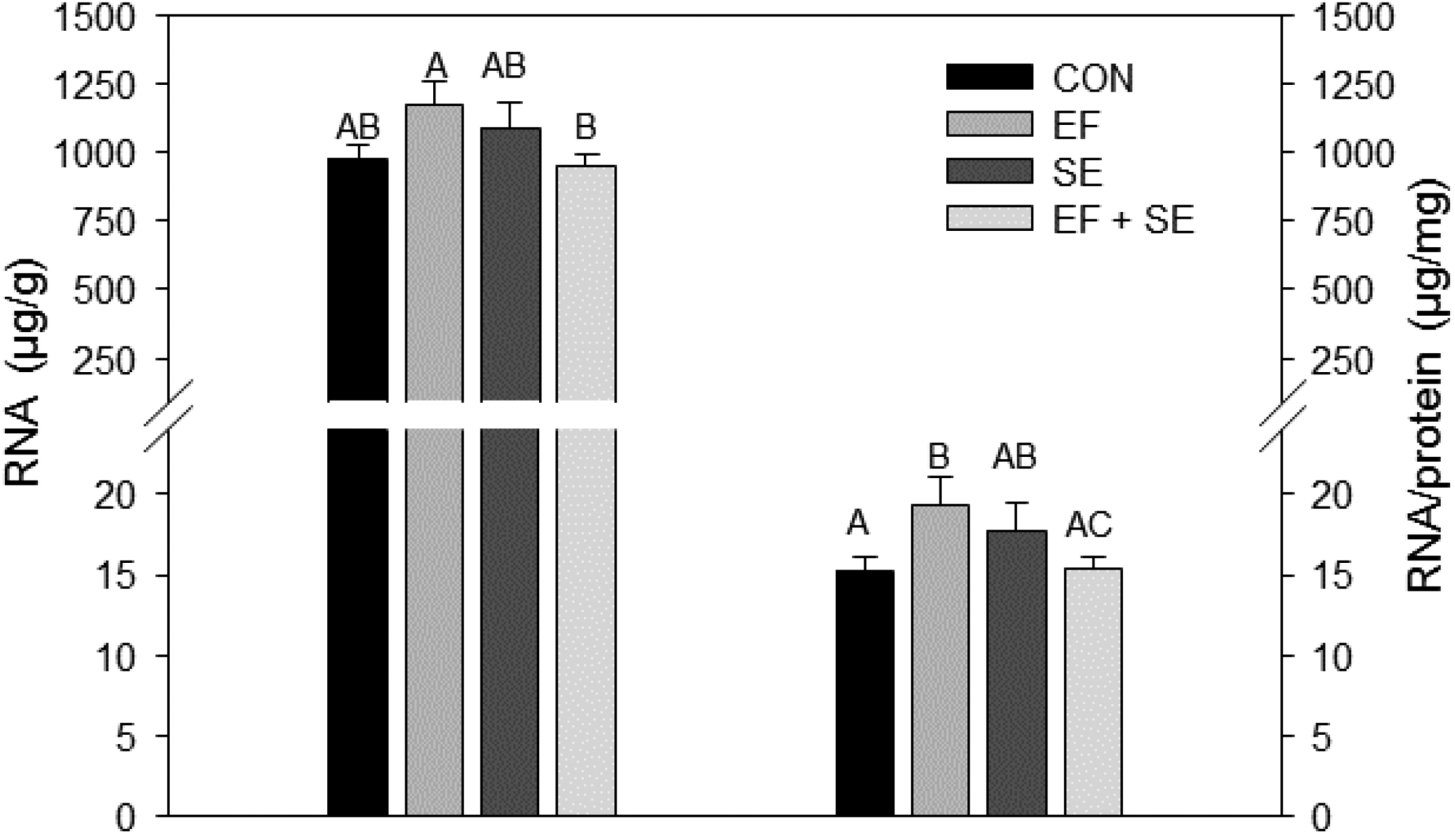
Pectoralis muscle RNA concentration and RNA/protein ratio at day 8 posthatching. Data are shown for control chicks (CON), and for chicks supplemented with the probiotic *Enterococcus faecium* AL41 from day 1 to day 7 of the experiment (EF), infected with *Salmonella* Enteritidis PT4 on day 4 of the experiment (SE) or that were administered with EF and SE (EFSE). Least square means and standard errors are represented by columns and error bars. Values not sharing a common superscript letter are significantly different (P = 0.006 for RNA; P = 0.01 for RNA/protein).

**Figure 2.**
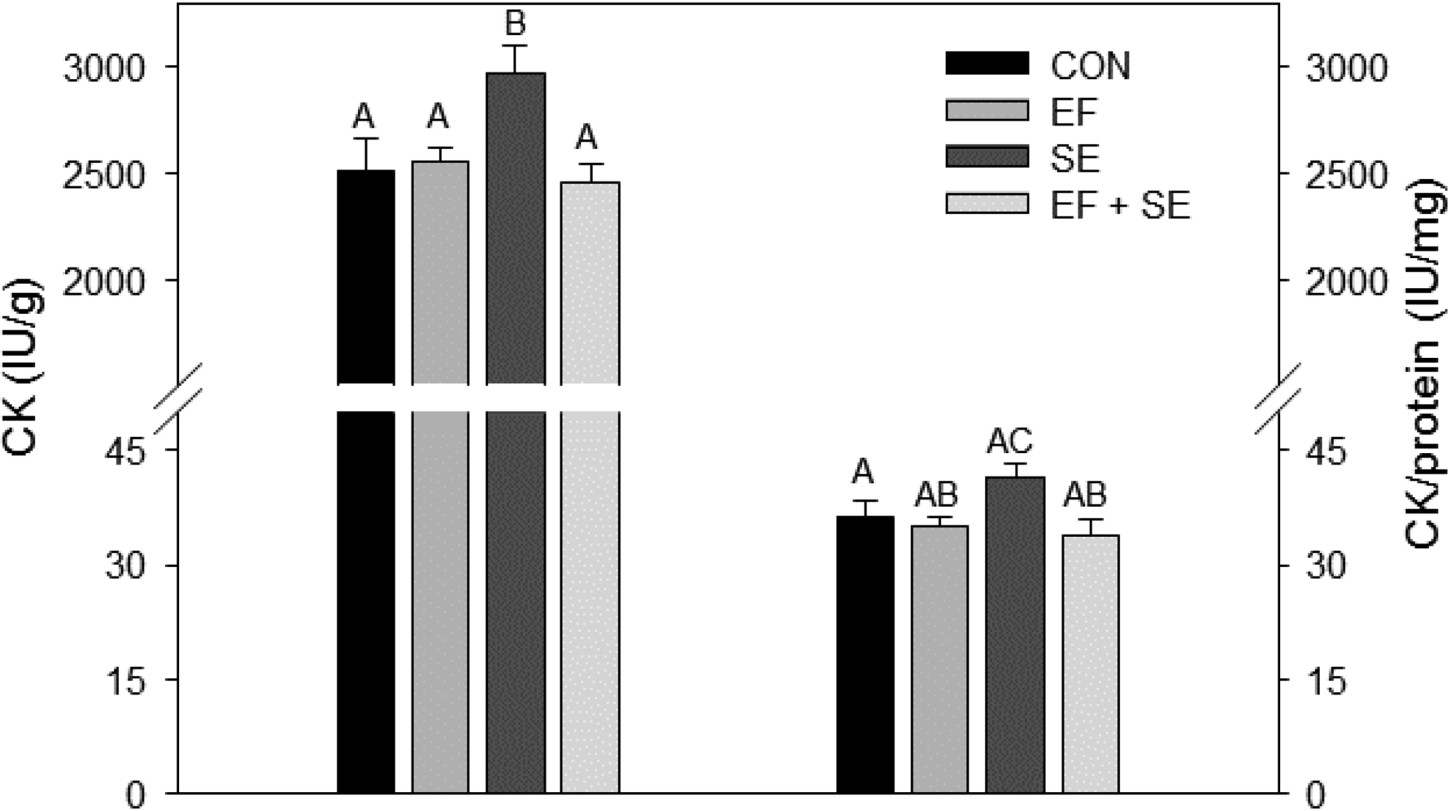
Pectoralis muscle creatine kinase (CK) concentration and CK/protein ratio at day 11 post-hatching. Data are shown for control chicks (CON), and for chicks supplemented with the probiotic *Enterococcus faecium* AL41 from day 1 to day 7 of the experiment (EF), infected with *Salmonella* Enteritidis PT4 on day 4 of the experiment (**SE**) or that were administered with EF and SE (EFSE). Least square means and standard errors are represented by columns and error bars. Values not sharing a common superscript letter are significantly different (P = 0.01).

Compared with chicks of the EFSE group, those supplemented with only EF had a higher RNA concentration in PM (D8). In addition, the RNA/protein ratio was higher in EF compared with CON and EFSE chicks at D8 (Figure 1). At D11, CK activity per g PM was higher in SE infected compared with CON, EF or EFSE birds. Related to muscle protein content, CK activity was higher in the SE group compared with the EF and EFSE groups (Figure 2).

### Pectoralis Muscle Microstructural Properties

Microstructural properties of the PM are summarized in Table 2. In all groups, FCSA and the number of capillaries per myofiber were higher on D11 compared with D8. The number of nuclei per myofiber increased with age, but this was significant for the EF and SE groups only. Total capillary area and CCSA increased in CON and EFSE groups, and capillary density rose in all groups except for EF.

**Table 2.** Microstructural properties of pectoralis muscle from untreated control chicks (**C**) and from chicks supplemented for seven days after hatching with *Enterococcus feacium* AL41 (EF), infected at day 4 of life with *Salmonella* Enteritidis PT4 (SE) or treated with both EF and SE (EFSE).

	Day 8				Day 11					P value	
	Control	EF	SE	EFSE	Control	EF	SE	EFSE	Age	Group	Age x Group
FCSA, μm^2^	347 ± 20^A^	246 ± 14^B^	262 ± 16^B^	289 ± 15^A,B^	581 ± 20^A^	533 ± 23^A^	448 ± 15^B^	461 ± 23^B^	<0.001	<0.001	0.01
Nuclei/fiber	0.79 ± 0.04^A^	0.66 ± 0.03^B^	0.64 ± 0.04^B^	0.72 ± 0.03^A,B^	0.81 ± 0.03	0.78 ± 0.03*	0.80 ± 0.03*	0.75 ± 0.03	*<0.001	0.035	0.08
CCSA, μm^2^	11.7 ± 0.7	10.3 ± 0.6	9.2 ± 0.7	10.3 ± 0.7	16.5 ± 1.3*^,A^	11.4 ± 0.8^B^	7.0 ± 0.5^C^	15.5 ± 0.6*^,A^	*<0.001	<0.001	<0.001
Capillary area, %	2.3 ± 0.1^A^	2.4 ± 0.2^A^	1.5 ± 0.1^B^	1.9 ± 0.2^A,B^	3.7 ± 0.2*^A^	2.3 ± 0.2^B^	1.6 ± 0.1^C^	3.7 ± 0.4*^A^	*<0.001	<0.001	<0.001
Capillary density, number per mm^2^	2002 ± 66^A,B^	2387 ± 104^A^	1737 ± 127^B^	1916 ± 87^A,B^	2413 ± 165*	2176 ± 130	2391 ± 147*	2612 ± 212*	*<0.001	0.377	0.004
Capillaries/fiber	0.79 ± 0.06^A^	0.70 ± 0.07^A,B^	0.52 ± 0.05^B^	0.58 ± 0.03^A,B^	1.25 ± 0.08	1.17 ± 0.06	1.01 ± 0.08	1.22 ± 0.07	<0.001	^#^0.002	0.49

Values are least square means ± standard error; FCSA, muscle fiber cross-sectional area; CCSA, capillary cross-sectional area.

For each time point (day 8 and day 11 of life), values not sharing a common superscript letter are significantly different.

*marks significant age-dependent changes of respective parameters within groups (C, EF, SE, EFSE);

#Control (*P* < 0.001) and EF (*P* = 0.01) vs. SE.

**Figure 3.**
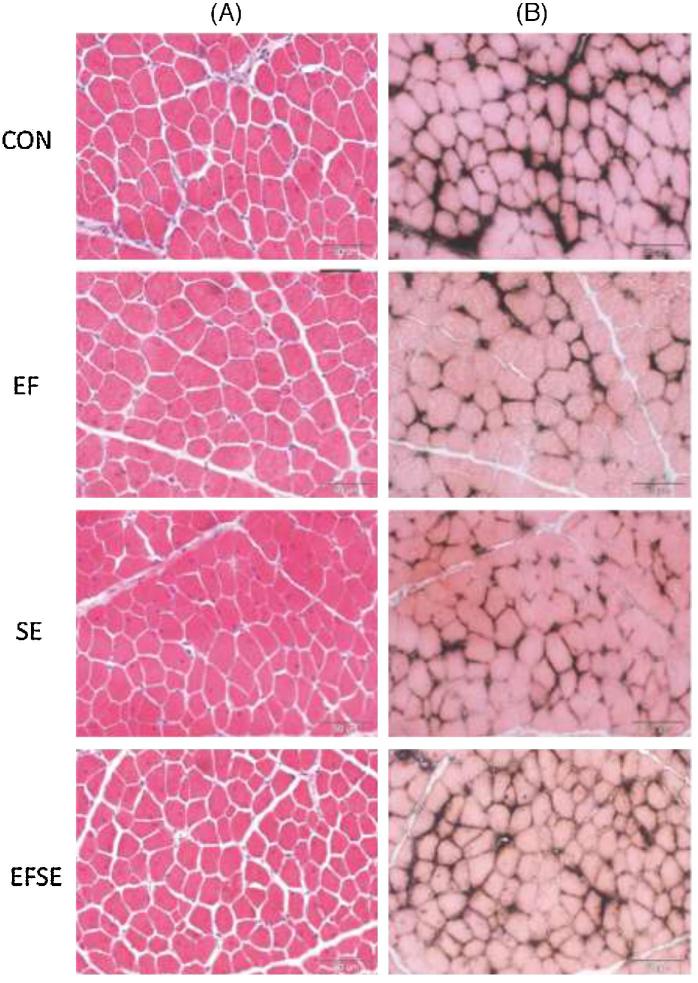
Morphological structure (**A**) and capillarization (**B**) of pectoralis muscle at day 11 post-hatching. Representative muscle tissue cross sections of control chicks (CON) and of chicks supplemented with the probiotic *Enterococcus faecium* AL41 from day 1 to day 7 of the experiment (EF), infected with *Salmonella* Enteritidis PT4 on day 4 of the experiment (SE) or that were administered with EF and SE (EFSE) are shown. Serial sections were stained with Hematoxilin/Eosin (A) to visualize nuclei (blue) or with Eosin-alkaline phosphatase (B) to visualize blood vessels (black) in addition to cytoplasm of myofibers (pink). Scale bars represent 50 μm.

Application of EF and infection with SE significantly reduced FCSA and nuclei per myofiber at D8 compared with controls. Figure 3 shows representative micrographs from PM of CON, EF, SE, and EFSE chicks at D11. At this time point, FCSA no longer differed between the CON and EF groups and was significantly higher than in SE and EFSE groups (Table 2, Figure 3).

PM capillarization was strongly affected by infection with SE (Table 2). A significant group effect revealed a reduced average number of capillaries per myofiber in SE-infected compared with CON and EF-supplemented birds. The area covered by capillaries (%) and the capillary density were both higher in the non-SE CON and EF groups at D8. Specifically, the capillary density was positively affected by probiotic supplementation leading to significant higher values in the EF compared with the SE group. Negative effects of SE infection on capillarization manifested at D11 as significant reduction of CCSA and the area (%) covered by capillaries (Table 2; Figure 3). Pre-feeding with EF completely abolished the negative effects of SE infection on capillarization in the EFSE group (Table 2; Figure 3). Nevertheless, CCSA and capillary area (%) were significantly lower in PM from EF chicks compared with the CON and EFSE groups.

## DISCUSSION

The first week posthatch is crucial for broiler PM growth (Halevy et al., 2000) and conditions or disturbances such as posthatch starvation or enteral infection can result in irreversible negative effects (Dina and Hams, 2016). Here, PM weight of control birds increased by 121% from 19 to 41 g within 3 D (D8 to D11). As myofiber formation is completed at hatching (Stockdale and Miller, 1987; Zhao et al., 2017), the observed PM weight gain relates mainly to fiber hypertrophy as reflected by higher FCSA and lower DNA/protein and RNA/protein ratios at D11 compared with D8 posthatching. In addition, upregulation of CK isoenzymes takes place during the development of skeletal muscle (Rehfeldt et al., 2010), as has previously been observed in studies with chicken (Bennett et al., 1985; Doherty et al., 2004). In the present experiment, total CK activity of PM increased by 28% from D8 to D11 thus also indicating a higher proportion of myofiber protein (Rehfeldt et al., 2010).

Substantial energy is required for such rapid growth and for various maturation processes in developing muscle, e.g., organization and time-dependent expression of contractile filaments, neuro-muscular structures, and capillaries and metabolic changes including the organization of mitochondrial structures and accompanying enzymes. Here, ICDH and LDH activities were measured as markers of oxidative and glycolytic muscle metabolism. In the predominantly fast twitch PM (Verdiglione and Cassandro, 2013), glycolysis is the main pathway through which energy is derived. In accord, LDH activity clearly dominates, and the age-dependent increase in LDH/ICDH ratio reflects the shift to the more glycolytic type of muscle metabolism characteristic for posthatch PM maturation (Kocamis et al., 2001; Doherty et al., 2004). This process is slowed down in EF-pre-treated birds infected subsequently with SE.

Both, the 7 D of pre-feeding with EF and the infection with SE suppress hypertrophic myofiber growth. However, in the EF group, negative effects (reduced FCSA and number of myonuclei per fiber) are completely compensated at D11, whereas the FCSA remains repressed at D11 in groups infected with SE (day 7 post infection) irrespective of whether EF was supplemented or not. Our results are in agreement with studies showing that any microbial colonization of the gut leads to reduced growth rates and declined feed efficiency attributable to local immune responses (Spurlock, 1997; Frost and Lang, 2008; Cook, 2011; Crhanova et al., 2011) and resistance to anabolic signals such as insulin, growth hormone, and IGF-1 (Frost et al., 2003; Haddad et al., 2005; Lang et al., 2005; Lorenzo et al., 2008; Whitlock et al., 2008). In a recent study, a disruption of main signalling pathways such as AMPK, insulin, and mTOR (mammalian target of rapamycin) that regulate important metabolic functions (Jacquemin et al., 2007) has been observed in PM of chicken infected with *Salmonella typhimurium* (Arsenault et al., 2013).

Although the immune system of chicks is immature during the first week of life, probiotic bacteria have been shown to activate and potentiate the innate immune system, i.e., macrophages and heterophiles, and to stimulate the adaptive immune response (Farnell et al., 2006; Crhanova et al., 2011; Karaffová et al., 2015). Specifically, EF has been shown to enhance IgA expression and secretion of IgA into the gut lumen, an effect that is potentiated and prolonged in SE-infected birds via transforming growth factor-ß4- and IL-17-dependent pathways (Karaffová et al., 2015). IgA helps to entrap antigens in the mucus and downregulates the expression of pro-inflammatory bacterial epitopes (Phalipon et al., 2002). In addition to its anti-inflammatory effect, EF supports gut villi development and, thus, the abilities for digestion and absorption in a positive way (Herich et al., 2010; Ševčíková et al., 2016). In contrast, a reduced proliferative activity of enterocytes, shorter villi in the jejunum and a decreased absorptive area (Herich et al., 2010; Ševčíková et al., 2016) have been found in SE-infected chicken, and these effects persist over longer time periods after infection. Thus, the nutritional and energetic status of SE-infected chicken will be reduced compared with control and EF supplemented birds.

Moreover, our data show that PM capillarization and, thus, delivery of O_2_, supply of nutrients, and removal of metabolites, are significantly lower in SE-infected chicks compared with CON and EF birds at both time points investigated. The increase of CK concentration and activity in PM, as observed in SE-infected chicken at D11, might thus be indicative of cellular energy stress (Wallimann et al., 1998; Schlattner et al., 2006). Sarcomeric mitochondrial (**MtCK**) and cytosolic CK enzymes are main components of the muscular phosphocreatine (**PCr**)-CK system (Jacobs et al., 1964; In ‘t Zandt et al., 1999). They catalyze transphosphorylation of intramitochondrial ATP into PCr and the reversible transfer of the N-phosphoryl group from PCr to ADP thereby regenerating and buffering cytosolic ATP levels (In ‘t Zandt et al., 1999; Schlattner et al., 2006). In addition, MtCK, which is present at high concentration in chicken PM (Bennett et al., 1985), is functionally coupled to glycolysis and facilitates the ATP/ADP exchange at the inner mitochondrial membrane (Schlattner et al., 2006). MtCK isoenzyme upregulation has specifically been observed in relation to cellular energy stress resulting, for example, from oxygen and glucose restriction, impaired mitochondrial function, or endotoxin or LPS challenges (Miller et al., 1993; Stadhouders et al., 1994; Hatano et al., 1996, 2004; O`Gorman et al., 1996, 1997; Heddi et al., 1999). Here, EF supplementation completely abolishes the CK upregulation, and in parallel, restores PM capillarization to control levels. Thus, increased CK activity in SE-infected chicken might be related to strong negative effects on capillarization of growing PM. This possibly represents a mechanism to compensate for a low cellular energy state by improving oxidative energy metabolism (Hatano et al., 1996; Schlattner et al., 2006), maintaining ATP levels, and preventing a drop of intracellular pH to abnormal low levels (Miller et al., 1993). In accord, creatine pyruvate feeding positively affects myofiber growth by increasing energy reserves of embryos *in ovo* and SC mitotic activity in neonatal broilers (Zhao et al., 2017).

During posthatch muscle growth, SC serve as the source of new myonuclei for myofiber growth (Jaquemin et al., 2007). In broiler chicken, mitotic activity of SC peaks as early as the first week after hatching when they are highly sensitive to nutritional signals (Halevy et al., 2000; Berri et al., 2006). The reduced number of nuclei per myofiber observed in EF and SE groups at D8 suggests suppression of SC proliferation in both groups followed by a significant compensatory increase until D11. Similar kinetics of SC proliferation has also been found in response to early posthatch feed deprivation (Halevy et al., 2000; Berri et al., 2006). In addition, immediate posthatch feed restriction (20%) induces an increase in MyoD expression also indicative of higher proliferation of muscle cells (Velleman et al., 2014). Nevertheless, FCSA was significantly smaller in both the SE and EFSE groups. When feed restriction was administered during the first week after hatching, a reduced expression of the early differentiation marker myogenin (Velleman et al., 2010, 2014) and of the neonatal myosin heavy chain isoform (Berri et al, 2006) has been observed, indicating a lowered ability of SC for differentiation. The processes of early and late differentiation are both mTOR-dependent (Erbay and Chen, 2001; Park and Chen, 2005), but this pathway is disrupted in PM of chicken infected by *Salmonella* Enteritidis (Arsenault et al., 2013). Thus, SC differentiation into mature muscle might be disturbed in SE-infected chicks, e.g., because of lack of appropriate signals (Berri et al, 2006; Jacquemin et al., 2007; Bryan et al., 2008; Velleman et al., 2010) and/or increased blood and tissue concentrations of bacterial cell wall components and pro-inflammatory cytokines (Al-Shanti et al., 2014; Castiglioni et al., 2015).

In contrast to EF feeding, the oral infection of young chicken with SE causes systemic infection (Levkut et al., 2009; Herich et al., 2010). In the liver, a major permissive site for intracellular proliferation and spread of SE into other organs and tissues (Vasquez-Torres et al., 1999), SE colonies have been found from day 4 to day 8 after oral infection (Herich et al., 2010). Moreover, muscle-resident (particularly macrophages) and infiltrating immune cells, muscle cells, and myofibers secrete pro-inflammatory cytokines (Bartoccioni et al., 1994; Pillon et al., 2013). IL-1, tumor necrosis factor-α, and interferon-γ have been shown to decrease the proliferation and differentiation of myoblasts (Spurlock, 1997; Castiglioni et al., 2015). Muscle infusion with a modest dose of IL-6 leads to atrophy reflected by a 9% decrease in total muscle protein and a 17% decrease in myofiber protein accompanied by reduced phosphorylation of S6K1 (Haddad et al., 2005).

Among the various extrinsic factors known to control SC behaviour, either via direct contact or by paracrine signals, microvascular cells play a central role (Abou-Khalil et al., 2009; Kostallari et al., 2015). Whereas endothelial cells have been shown to stimulate SC proliferation, associated pericytes, which are embedded into the capillary basal lamina, promote the SC differentiation program and are important for angiogenesis, microvasculature structural integrity, and blood flow regulation (Abou-Khalil et al., 2009; Armulik et al., 2011; Kostallari et al., 2015). Thus, to ensure normal muscle development, tissue vascularization, which is rudimentary at hatching, must develop in parallel to myogenesis (Hoving-Bolink et al., 2000) and both processes are regulated by common factors, with VEGF (vascular endothelial growth factor) as a master driver (Coultas et al., 2005; Christov et al., 2007; Bryan et al., 2008). Our results reveal strong negative effects of SE-infection on the capillarization of growing PM. Specifically, the CCSA, the area (%) covered by capillaries, and the number of capillaries per myofiber are reduced compared with CON and EF-supplemented birds. Thus, early SE infection might not only stress the muscle energy metabolism, but also reduce the functionality of the microvascular cells, SC and myoblasts irreversibly to impair muscle growth.

In conclusion, our study of broiler chicks reveals negative effects of early SE-infection on PM maturation, hypertrophic growth, and capillarization, all of which can contribute to lower growth performance and meat quality (Dransfield and Sosnicki, 1999; Halevy et al., 2000; Hoving-Bolink et al., 2000). Energetic stress and a reduced functionality of the main cell types (SC, myoblasts, microvascular cells) during the critical first weeks posthatching (Halevy et al., 2000, 2004) are possible causes. Preventive feeding of the probiotic strain EF AL41 restores PM capillarization and, therefore, reduce energetic stress by ameliorating the delivery of O_2_, the supply of nutrients, and the removal of metabolites. Supplementation of EF AL41 might therefore be a useful tool for improving muscle metabolism during SE infection and, thus, broiler performance and meat quality (Al-Khalaifah, 2018).

## Supplementary Material

Supplemental File
