# Supplementary material for: Muscle characteristics in chicks challenged with Salmonella Enteritidis and the effect of preventive application of the probiotic Enterococcus faecium
Source: Poult Sci. 2018 Dec 26;98(5):2014–25. doi: 10.3382/ps/pey561 (PMC6448134; doi:10.3382/ps/pey561)
Supplement: Supplemental File [file pey561_supplemental_file.docx]

Table 1. Weight, contents and concentrations of DNA, RNA, and protein, and enzyme activities in pectoralis muscle (**PM**) from untreated control chicks (**C**), and from chicks supplemented for seven days after hatching with *Enterococcus feacium* AL41 (**EF**), infected at day 4 of life with *Salmonella* Enteritidis PT4 (**SE**) or treated with both EF and SE (**EFSE**)

|  | Day 8 | | | | Day 11 | | | | P value | | |
| --- | --- | --- | --- | --- | --- | --- | --- | --- | --- | --- | --- |
|  | C | EF | SE | EFSE | C | EF | SE | EFSE | Age | Group | Age x Group |
| DNA, µg/mg | 161±3 | 174±5 | 166±14 | 158±9 | 145±3 | 150±6* | 141±4* | 163±7 | *0.006 | 0.467 | 0.152 |
| RNA, µg/mg | 972±57^AB^ | 1174±80^A^ | 1088±97^AB^ | 949±44^B^ | 660±24 | 739±27 | 758±35 | 785±28 | <0.001 | 0.068 | 0.121 |
| Protein, mg/g | 64±1.2 | 62±2.0 | 62±1.6 | 62±1.2 | 69±2.6 | 73±2.1* | 72±2.2* | 73*±3.4 | *<0.001 | 0.964 | 0.420 |
|  |  |  |  |  |  |  |  |  |  |  |  |
| DNA/protein, µg/mg | 2.52±0.06 | 2.85±0.14 | 2.70±0.25 | 2.56±0.18 | 2.10±0.06* | 2.06±0.04* | 1.97±0.06* | 2.23±0.07 | *<0.001 | 0.689 | 0.213 |
| RNA/protein, µg/mg | 15.2±1.0^A^ | 19.3±1.7^B^ | 17.7±1.7^AB^ | 15.3±0.8^AC^ | 9.6±0.7 | 10.2±0.5 | 10.5±0.3 | 10.8±0.5 | <0.001 | 0.122 | 0.146 |
| protein/RNA, mg/µg | 0.067±0.004 | 0.054±0.006 | 0.060±0.007 | 0.066±0.004 | 0.106±0.007 | 0.099±0.005 | 0.096±0.003 | 0.094±0.004 | <0.001 | 0.250 | 0.414 |
|  |  |  |  |  |  |  |  |  |  |  |  |
| CK IU/g | 2067±81 | 1927±75 | 2093±95 | 2085±45 | 2509±152^A^ | 2549±75^A^ | 2972±129^B^ | 2455±87^A^ | <0.001 | 0.016 | 0.058 |
| CK/protein, IU/mg | 32.2±0.9 | 31.3±0.5 | 34.0±1.7 | 33.6±0.70 | 36.3±2.0^A^ | 35.0±1.4^AB^ | 41.4±1.8*^AC^ | 33.9±2.2^AB^ | *<0.001 | 0.023 | 0.151 |
| ICDH, IU/g | 2.49±0.06 | 2.57±0.05 | 2.53±0.11 | 2.50±0.07 | 2.30±0.13 | 2.34±0.12 | 2.36±0.06 | 2.47±0.15 | 0.028 | 0.842 | 0.742 |
| LDH, IU/g | 478±35 | 417±36 | 415±18 | 458±30 | 619±38* | 648±47* | 653±33* | 552±21 | *<0.001 | 0.624 | 0.103 |
| ICDH/protein,IU/mg | 0.039±0.001 | 0.042±0.002 | 0.041±0.002 | 0.040±0.001 | 0.033±0.002 | 0.032±0.001 | 0.033±0.001 | 0.034±0.001 | <0.001 | 0.944 | 0.551 |
| LDH/protein, IU/mg | 7.46±0.56 | 6.73±0.35 | 6.74±0.35 | 7.40±0.55 | 8.97±0.57* | 8.85±0.51* | 9.12±0.54* | 7.63±0.52 | *<0.001 | 0.569 | 0.156 |
| LDH/ICDH | 192±14 | 163±16 | 166±10 | 184±12 | 275±27* | 281±27* | 278±17* | 227±15 | *<0.001 | 0.500 | 0.171 |

Values are least square means ± standard error. CK, creatine kinase; ICDH, isocitrate dehydrogenase; LDH, lactate dehydrogenase. For each time point (day 8 and day 11 of life), values not sharing a common superscript letter are significantly different (P < 0.05); *marks significant (P < 0.05) age-dependent changes of respective parameters within groups (C, EF, SE, EFSE).
